# The Story of the Insane from Year to Year

**Published:** 1895-04-06

**Authors:** 


### THE STORY OF THE INSANE FROM YEAR TO YEAR.

(7th Series.)

### CHESHIRE COUNTY ASYLUM, PARKSIDE.

On January 1st this asylum contained 661 patients, and on the last day of the year the number had risen to 705. The admissions were 240, the recoveries were 69, and the deaths 76. We also notice that 22 cases were discharged "relieved." The recoveries stand at 32·2 per cent. on the admissions, a percentage considerably below the average of other asylums, and also below their own average, which stands at 40 4. Dr. Sheldon explains the low rate as being due to the unfavourable nature of the admissions, and he truly adds that as a test of the efficiency of an asylum the rate of recovery is often likely to be fallacious. Turning to Table X., we find that 20 of the admissions were driven insane by domestic trouble and adverse circumstances, while drink was the cause in 29, and hereditary influence in 31. The deaths work out at 11 per cent. on the average number resident. Consumption caused death in 13 instances. Although the number of inmates increased by 44 during the year, it is consoling to find that Dr. Sheldon does not attribute the increase to an increased production of insanity. Medical superintendents have almost ceased to write about the New Lunacy Act, regarding it probably as one of those evils which must be endured as it cannot be cured; and it is only when re-certification is forgotten, or is impossible, that reference to the Act crops up in asylum reports. Dr. Sheldon had three such cases, and he thinks that this defect in the Act needs amending. No doubt it does, but we think it would be better to sweep the greater part of the Act away and begin *de novo*. Like many other superintendents, Dr. Sheldon drops a tear over Mr. Cleaton's retirement from the Board of Lunacy. Ann Voer received a pension of £25 a year, and Mrs. Hickin, the housekeeper, received one of £50, but neither of these lived long to enjoy it. The engineer was granted £40 a year as pension, and it is to be hoped he will be more fortunate than his late colleagues. The weekly cost per head is 9s.

### BIRMINGHAM BOROUGH ASYLUM, WINSON GREEN.

During last year the numbers rose from 619 to 624. The admissions were 374, the recoveries 163, and the deaths 104. Calculated in the usual way the percentage of recoveries stands at 43·5, and of deaths at 16·6, both of these percentages being considerably above the average. In addition to the recoveries 23 patients were discharged relieved. Of the admissions, 38 were owing to drink and 47 to hereditary influences. In 149 cases the cause of insanity was unknown. Brain diseases caused death in 51 cases and consumption in 9. Mr. Whitcomb has set apart, on each side of the asylum, a ward for recent cases, and he keeps all these cases in bed for some time after admission. This was the system pursued by some of the older asylum superintendents a generation since, and it has unquestionably much in its favour, and as Mr. Whitcomb expresses, it gives opportunity for "improved clinical observation by the medical officers." The asylum would seem to be somewhat crowded; but Mr. Whitcomb does not think any injurious effect upon the health of the patients has taken place. Four of the attendants and nine of the nurses have passed the examination of the Medico-Psychological Association and received certificates of proficiency in nursing. The weekly charge per head is, for borough patients, 9s.; and for private patients, from 10s. to 30s.

### THE WHITTINGHAM ASYLUM, PRESTON.

The number of patients increased from 1,855 to 1,865. There were 407 admission, 160 recoveries, and 204 deaths. The percentage of recoveries on the admissions was 39·31, and that of the deaths on the average number resident was 10·96. Not fewer than 103 of the admissions were driven mad by intemperance in drink, and hereditary influence accounts for 86. Cerebral disease in one form or other caused death in 86 cases, and consumption in 32. A considerable part of Dr. Wallis' report is taken up with a statement of his views relative to the treatment of acute cases of insanity in detached hospitals, and he looks for great results from it, believing that too much routine work and the limited number of medical officers have smothered research. "I am," he says, "most hopeful that the movement which is setting in for the establishment of small separate hospitals in asylums will be productive of much progress, much enlightenment." Few people have had more experience in making post-mortem examinations than Dr. Wallis; and we find him saying, "The usual pathological work in asylums is so much confined to the examination of the utterly wasted and degenerated brains of the general paralytics chronically disorganised and senile brains, confirmed, epileptics. What we more particularly want is the careful and exhaustive examination by many workers of any brain which may be available from the unexpected, sudden, or early death of a general paralytic in the first stage; a recent case of melancholia or acute mania; dying from pneumonia or some sudden complication or accidental circumstances." In all this we fully agree with Dr. Wallis, and we hope we may interpret the first sentence as the beginning of a well-timed reaction against the making of post-mortem examinations in cases where no good end can be served. There are many other interesting remarks which want of space forbids extraction, but we cannot refrain from giving prominence to the following. "Experience has taught us that airing court walls are for the most part mischievous, and seem to furnish a direct incentive to attempts at escape." The abolition of airing court walls and the introduction of unlocked doors have been gradually feeling their way in the asylums of Scotland, and as Dr. Wallis is now a Commissioner in Lunacy for England, it is greatly to be desired that these parts of the Scottish administration of asylums will be engrafted on our English ones. The weekly cost of maintenance is 9s. 1d. per head.

